# Construction of a Miniaturized Chromatic Acclimation Sensor from Cyanobacteria with Reversed Response to a Light Signal

**DOI:** 10.1038/srep37595

**Published:** 2016-11-24

**Authors:** Mitsuharu Nakajima, Stefano Ferri, Matthias Rögner, Koji Sode

**Affiliations:** 1 Department of Biotechnology and Life Science, Graduate School of Engineering, Tokyo University of Agriculture & Technology, 2-24-16 Naka-cho, Koganei, Tokyo 184-8588, Japan; 2 JST, CREST, 2-24-16 Naka-cho, Koganei, Tokyo, 184-8588, Japan; 3 Department of Applied Chemistry and Biochemical Engineering, Shizuoka University, 3-5-1 Johoku, Naka-ku, Hamamatsu, Shizuoka, 432-8561, Japan; 4 Plant Biochemistry, Faculty of Biology & Biotechnology, Ruhr-Universität Bochum, Universitätsstr. 150, 44780, Bochum, Germany; 5 Institute of Global Research Innovation, Tokyo University of Agriculture & Technology, 2-24-16 Naka-cho, Koganei, Tokyo 184-8588, Japan

## Abstract

Cyanobacteria harbor unique photoreceptors, designated as cyanobacteriochromes (CBCRs). In this study, we attempted to engineer the chromatic acclimation sensor CcaS, a CBCR derived from the cyanobacterium *Synechocystis* sp. PCC 6803. The wild-type CcaS induces gene expression under green light illumination and represses it under red light illumination. We focused on the domain structure of CcaS, which consists of an N-terminal transmembrane helix; a GAF domain, which serves as the sensor domain; a linker region (L1); two PAS domains; a second linker region (L2); and a C-terminal histidine kinase (HK) domain. Truncated versions of the photoreceptor were constructed by removing the L1 linker region and the two PAS domains, and fusing the GAF and HK domains with a truncated linker region. Thus constructed “miniaturized CcaSs” were grouped into four distinct categories according to their responses toward green and red light illumination, with some showing improved gene regulation compared to the wild type. Remarkably, one of the miniaturized CcaSs induced gene expression under red light and repressed it under green light, a reversed response to the light signal compared to wild type CcaS. These characteristics of engineered photoreceptors were discussed by analyzing the CcaS structural model.

Recent advances in our understanding of photosensing in biological systems have led to the use of photoreceptors as novel genetic tools that regulate gene expression in bioprocess models. Optogenetics, which uses various photoreceptors to directly control cell behaviors via light exposure, has recently attracted attention as a synthetic biology-based bioprocess design. Light-regulated microbial bioprocesses using photoreceptors have been reported, demonstrating that gene expression can be regulated without the addition of chemical inducers to the cultures^1^,^2^,^3^,^4^,^5^.

Among several organisms, cyanobacteria are the most attractive sources of photoreceptors. Most cyanobacteria are capable of modulating the biosynthesis of phycobilisome or inducing phototaxis in response to light conditions. These bacteria harbor unique photoreceptors known as cyanobacteriochromes (CBCRs). CBCRs are further categorized into several subclasses^6^,^7^,^8^,^9^,^10^,^11^,^12^,^13^,^14^ based on their primary sequences, spectral properties, and chromophores, which can sense green/red light, red/green light, ultraviolet-A, blue/green light, and far red/green light. Some CBCRs belong to two-component regulatory systems, inducing phenotypic changes such as phototaxis and the expression of light harvesting proteins for photosynthesis^15^,^16^,^17^,^18^,^19^,^20^.

Among several CBCRs, we focused on the optogenetic application of the chromatic acclimation sensor CcaS from the unicellular cyanobacterium *Synechocystis* sp. PCC 6803. CcaS has a phycocyanobilin (PCB) chromophore and is part of a green/red light sensing two-component regulatory system. CcaS is a sensor histidine kinase (HK), and together with its cognate response regulator CcaR, chromatically regulates the expression of the phycobilisome linker gene *cpcG2*. CcaS regulates the phosphorylation of CcaR, which when phosphorylated, promotes gene expression by binding to the P_*cpcG2*_ promoter region. Gene expression is promoted by the CcaS-CcaR two-component system under green light exposure and repressed under red light^20^.

The construction of a green light-regulated recombinant gene expression system in *Synechocystis* sp. PCC 6803 has been previously reported^21^. The system was then applied to regulate T4 phage-derived lysis genes in the construction of a green light-inducible autolysis system for cyanobacteria^3^. In addition, CcaS, CcaR, and P_*cpcG2*_ from *Synechocystis* sp. PCC 6803 have been introduced into the marine cyanobacterium *Synechococcus* sp. NKBG 15041c to achieve green light-regulated recombinant gene expression in this strain^22^. Recently, CcaS-based gene regulation of the autotransporter protein Antigen43 (Ag43) was employed in the construction of a cell recovery system for non-photosynthetic microorganisms, such as *Escherichia coli*^5^. Although these findings highlight the great possibilities in employing CcaS-based green light sensing systems as novel recombinant gene expression tools in bioprocesses, the regulation of gene expression levels by CcaS is not precise and the background expression level under non-inductive, red light illumination is not negligible. In addition, altering the direction of gene expression (i.e., gene induction under red light and repression under green light) expands the application of this system. Therefore, further engineering studies are needed to improve CcaS-directed gene regulation.

CcaS consists of the following domains and regions: an N-terminal transmembrane helix, a sensor domain consisting of a cyanobacteriochrome-type cyclic guanosine monophosphate phosphodiesterase/adenylyl cyclase/formate hydrogen lyase transcriptional activator (GAF) domain, a linker region (L1), two period/aryl hydrocarbon receptor nuclear translocator/single-minded (PAS) domains of unknown function, a second linker region (L2), and a C-terminal HK domain (Fig. 1). Hirose *et al.* previously reported that light exposure lead to a reversible absorption spectrum change in the GAF domain, subsequently inducing autophosphorylation of the HK domain, followed by transfer of the phosphoryl group to the cognate response regulator CcaR^20^, which in turn binds to P_*cpcG2*_to promote the transcription of the *cpcG2* gene.

In this study, the engineering of the cyanobacterial photoreceptor CcaS was carried out focusing on the domain structures of the protein. Several truncated CcaSs were constructed and their sensor HK properties were investigated. The aim of the study was to develop a photoreceptor capable of tight gene regulation under light exposure. The truncated CcaSs were constructed by removing two PAS domains and fusing together the GAF and HK domains with a truncated linker region, to form “miniaturized CcaSs”. Our findings reveal that the constructed miniaturized CcaSs displayed enhanced gene expression-regulation abilities. In addition, one of the miniaturized CcaSs showed a reversed response, inducing gene expression under red light and repressing under green light. The properties of these miniaturized CcaSs are discussed based on the postulated structural motifs.

## Results

### Design and Construction of Miniaturized CcaSs

**Figure 1:**
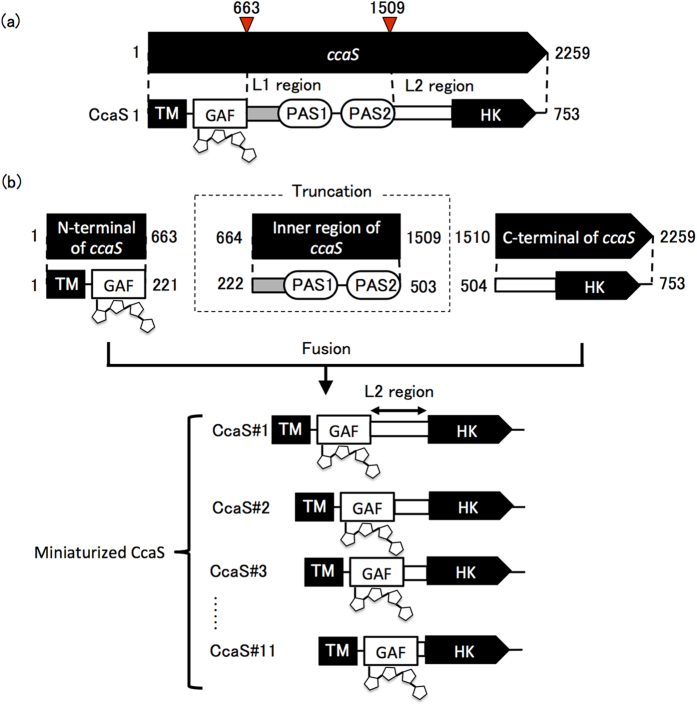
Domain organization of wild-type CcaS (**a**) and miniaturized CcaSs **(b).** Miniaturized CcaSs were constructed by deleting the region encoding the L1 region and the two PAS domains (amino acids 222–503). The region encoding the GAF domain was fused in-frame to the L2 linker region, with an incremental number of residues removed from the N terminus of the L2 linker.

Various miniaturized CcaSs were created by preparing the corresponding structural genes as follows. The region of the *ccaS* gene encoding the L1 linker region and the two PAS domains (amino acids 222 to 503, encoded by nucleotides 664 to 1509) was deleted. The region encoding the GAF domain was connected to the HK domain by fusing it in-frame to L2 linker region (Fig. 1a,b). A range of truncated CcaSs, designated CcaS#1 to CcaS#11, were prepared by deleting an incremental number of amino acids from the N terminus of the L2 linker region.

### Characterization of the Miniaturized CcaSs

The constructed miniaturized CcaSs were functionally expressed in *E. coli* together with CcaR, and the genes necessary for the synthesis of PCB. The miniaturized CcaSs were characterized by measuring the expression level of the red fluorescent protein (RFP) gene, which was inserted downstream of the P_*cpcG2*_ promoter and whose expression is expected to be induced when CcaR is activated by the miniaturized CcaSs. The transformants were cultivated under either red or green light illumination and their sensor responses were evaluated and compared to those of the transformant containing the wild-type CcaS.

**Figure 2:**
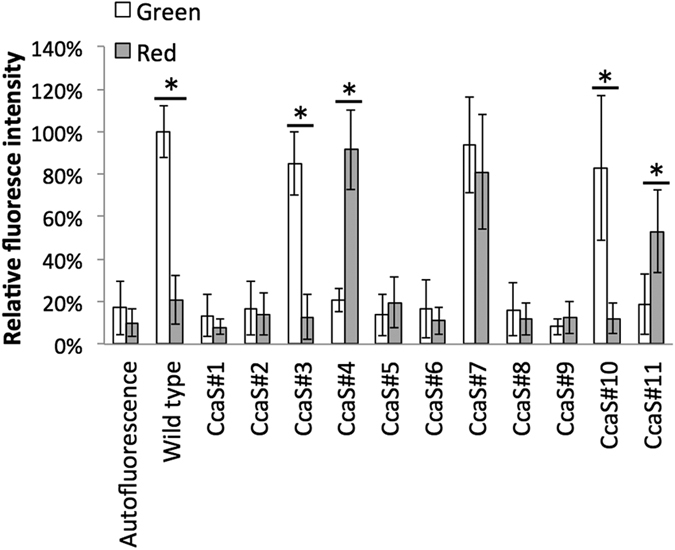
Characterization of the miniaturized CcaSs by normalized fluorescence level. Bars show the fluorescence of cells grown under green light (white bars) or red light (gray bars). Bars indicate relative fluorescence intensity to normalized fluorescence level of wild type. Data represent means ± SD from independent triplicate experiment from each of three clones (nine experiments). Asterisks indicate statistically significant differences between fluorescence level of cells under green light and red light. (Dunnet’s test; *P < 0.001).

Among the transformants harboring the 11 miniaturized CcaSs, six (CcaS#1, CcaS#2, CcaS#5, CcaS#6, CcaS#8, and CcaS#9) showed no or negligible fluorescence levels under both red or green light illumination (Fig. 2). These miniaturized CcaSs were therefore either inactively produced or are in a constant state of repression of gene expression. Five miniaturized CcaSs (CcaS#3, CcaS#4, CcaS#7, CcaS#10, and CcaS#11) showed RFP-derived fluorescence under various light conditions. The CcaS#3 and CcaS#10 transformants displayed similar high fluorescence intensities to wild-type CcaS under green light illumination and repressed levels under red light illumination. Therefore, miniaturized CcaS#3 and CcaS#10 showed similar gene regulation pattern as wild-type CcaS. Under both green and red light illumination, the CcaS#7 transformant displayed similarly high fluorescence levels as wild-type transformants grown under green light. Therefore, unlike wild-type CcaS, miniaturized CcaS#7 was constantly in an activated state. Interestingly, the transformant harboring CcaS#4 and CcaS#11 displayed fluorescence only under red light illumination, but it was repressed under green light. Therefore, the miniaturized CcaS#4 and CcaS#11 showed the opposite regulation pattern compared the wild-type CcaS, although both harbor the same PCB chromophore.

The observed fluorescence levels under non-induced conditions of the transformants harboring miniaturized CcaSs were comparable to the autofluorescence of *E. coli* without the RFP structural gene and considerably less (~50%) than the level of the wild-type CcaS transformant. These results indicate that the background expression levels of the gene under the regulation of miniaturized CcaSs were more strictly repressed than with the wild-type CcaS. To examine possible additional applications, the miniaturized CcaSs that showed either green light or red light gene induction (CcaS#3, CcaS#10, and CcaS#11) were further characterized.

### Green or Red Light-Regulated Gene Expression Using Miniaturized CcaSs

All transformants showed indistinguishable cell growth from those harboring wild-type CcaS or lacking the RFP gene (Supplementary data, Fig. S1). Therefore, the expression of the miniaturized or wild-type CcaS had no detectable effect on cell growth.

**Figure 3:**
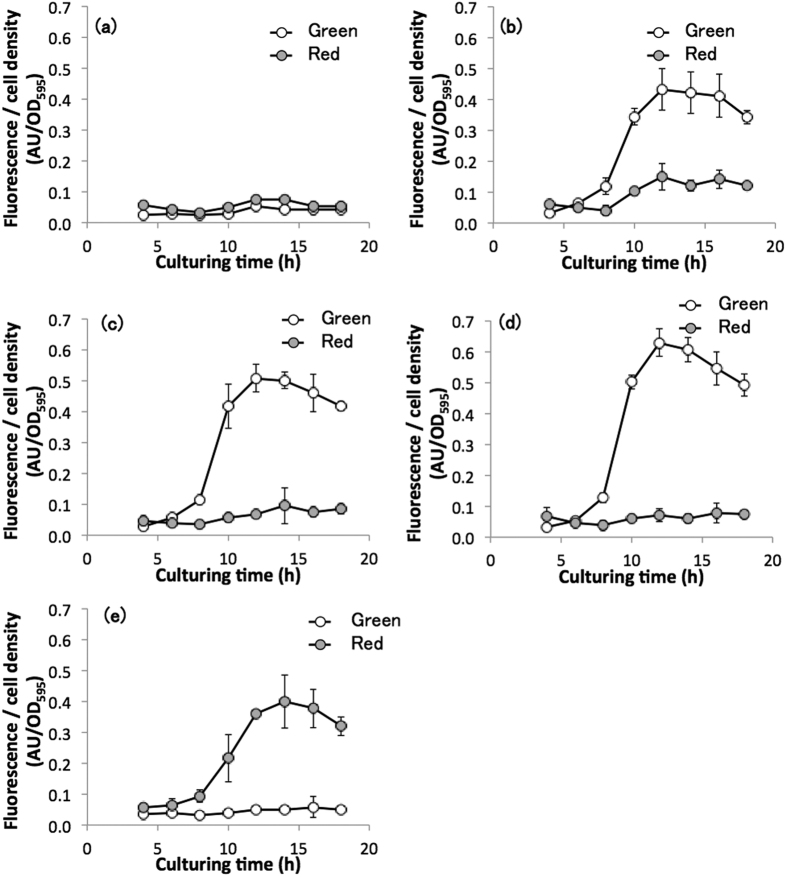
Green or red light-regulated gene expression using miniaturized CcaSs. Fluorescence was measured in cells cultured under either green (white circles) or red (gray circles) light exposure. Autofluorescence was measured in cells harboring a light sensing system with the wild-type CcaS and without the *rfp* gene (**a**). Characterization of CcaSs was carried out in cells harboring the *rfp* gene and a light sensing system with either the wild-type CcaS (**b**), CcaS#3 (**c**), CcaS#10 (**d**), or CcaS#11 (**e**). Data represent means ± SD from independent triplicate experiment from one clone (three experiments).

Representative time courses of normalized RFP expression levels of transformants containing the various miniaturized CcaS or wild-type CcaS are shown in Figure 3a to e. Under green light illumination, the transformant harboring wild-type CcaS showed a clear increase in RFP fluorescence intensity after 8 hours of cultivation (Fig. 3b). The maximum RFP expression level (0.4 AU) was observed after 12 hours of cultivation. Under red light illumination, an augmentation in RFP expression was also observed after 10 hours of cultivation, and the maximum expression peaked at 12 hours. The RFP expression level under red light was about 0.1 AU; this is 25% of the level observed under green light. However, the repressed fluorescence level under red light illumination was more than 2-fold the intensity observed by auto-fluorescence (Fig. 3a). Therefore, these results confirmed that wild-type CcaS induced the gene expression of the P_*cpcG2*_–linked gene under green light and repressed it under red light illumination; however, the repression level was incomplete and leakage of the gene expression was observed.

Figure 3c and d show the time course of RFP expression for miniaturized CcaS#3 and CcaS#10, respectively. In both transformants, RFP expression levels increased after 8 hours of cultivation (Fig. 3c and d, respectively) under green light illumination. The maximum RFP intensity was observed after 12 hours of cultivation. The peak RFP expression levels of transformants harboring CcaS#3 and CcaS#10 were similar or higher than those achieved by transformants harboring wild-type CcaS. In contrast, under red light illumination, no obvious RFP expression was observed, with fluorescence levels comparable to that of *E. coli* autofluorescence (Fig. 3a). Therefore, these results indicate that miniaturized CcaS#3 and CcaS#10 induced the gene expression of the P_*cpcG2*_–linked gene under green light at a similar level of wild type, and almost completely repressed the gene expression under the red light illumination.

The regulation of RFP expression in the miniaturized CcaS#11 transformant was the complete reverse of those harboring the wild-type CcaS, miniaturized CcaS#3, and miniaturized CcaS#10. The miniaturized CcaS#11 transformant showed an increase in RFP expression levels after 8 hours of cultivation (Fig. 3e) under red light illumination. Under green light illumination, no obvious RFP expression was observed, with fluorescence levels comparable to the *E. coli* autofluorescence (Fig. 3a). The maximum RFP expression level (0.4 AU), observed after 12 hours of cultivation under red light, was about 90% of that observed in the wild-type CcaS transformant under green light. Therefore, these results indicate that miniaturized CcaS#11 induced the expression of the P_*cpcG2*_–linked gene under red light at a comparable level as the wild-type CcaS under green light, and almost completely repressed gene expression under green light illumination.

Evaluation of gene expression under green or red light illumination has demonstrated that the three active miniaturized CcaSs showed almost similar inducing abilities, comparable to the wild-type CcaS (summarized in Supplementary data Figure S2). Furthermore, the miniaturized CcaSs showed much strict repression compared to wild-type CcaS, with no increase in fluorescence above the background *E. coli* autofluorescence. Interestingly, the miniaturized CcaS#11 showed a completely reversed response, inducing and repressing gene expression under red and green light, respectively. These findings indicate that engineered CcaS were successfully developed by truncating the PAS domain and modifying the length of the L2 region, thus constructing miniaturized CcaSs capable of high expression levels under inductive conditions and very strict repression of gene expression under non-inductive conditions.

## Discussion

In this study, miniaturized CcaSs were engineered by deleting the two PAS domains and the L1 region, as well as deleting an incremental number of amino acid residues from the N terminus of the L2 linker region. Thus constructed miniaturized CcaSs showed a wide range of responses to light and tightly regulated gene expression in comparison to the wild-type CcaS. In the CcaS/CcaR two-component regulatory system, gene expression is regulated by phosphorylation of CcaR, which is regulated by autophosphorylation of CcaS. CcaS autophosphorylation level increases under green light exposure and decreases under red light exposure. The phosphoryl group is then transferred from the phosphorylated CcaS to CcaR^21^. The molecular mechanism of CcaS autophosphorylation, however, remains unknown.

Möglich and colleagues reported the construction of a blue light sensor protein, YF1, a chimeric protein that is composed of the light sensor domain of YtvA from *Bacillus subtilis* and the HK domain of oxygen sensor protein FixL from *Bradyrhizobium japonicum*^23^. The crystal structure of YF1 has been elucidated^24^ and the screening of a YF1 mutant library identified YF1 mutants showing different gene expression induction abilities and distinct responses to light signals^25^. The structure of YF1 suggests that the α-helix linker region connected to the HK domain rotates about 40°–60° when the light sensor domain is exposed to the light signal^23^. The rotation modulates the interaction between the active site His residue in the HK domain and the adenosine triphosphate (ATP) binding site, thereby triggering autophosphorylation at of the His residue — known as the rotary-switch mechanism^23^.

The miniaturized CcaSs were grouped into four categories base on their characteristics: Type 1 showed similar gene regulation patterns as wild-type CcaS, inducing gene expression under green light illumination and repressing it under red light illumination (CcaS#3 and CcaS#10); Type 2 induced gene expression under both green and red light illumination (CcaS#7); Type 3 showed the opposite gene regulation pattern to wild-type CcaS, inducing gene expression under red light illumination and repressing it under green light (CcaS#4 and CcaS#11); and Type 4 showed no gene induction under any light conditions (CcaS#1, CcaS#2, CcaS#5, CcaS#6, CcaS#8, and CcaS#9).

**Figure 4:**
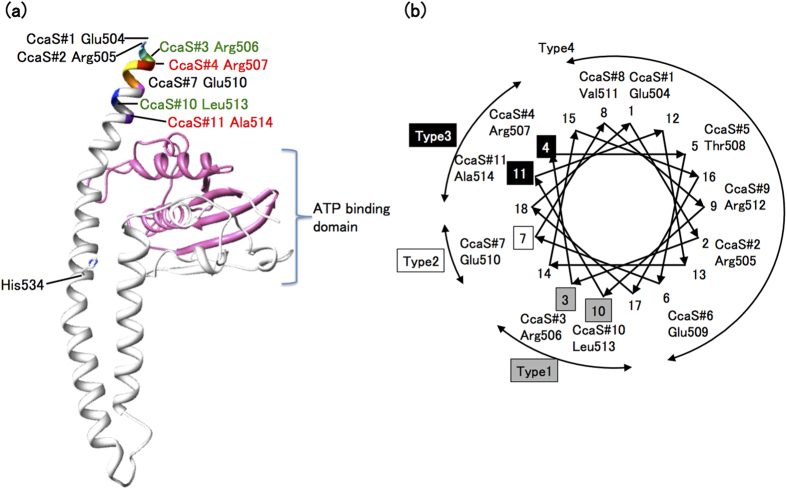
The structural model of 504–753 amino acid region of CcaS (**a**) and the α-helical wheel model of the L2 region (**b**). The number appeared in the α-helical wheel model (1–18) are the helical register number.

To increase our understanding of the photoregulation of CcaS, a structural model of the C-terminal region of CcaS was constructed (Fig. 4a). This model contains the region including residues 504–753 of the L2 region and the HK domain of CcaS based on the secondary structure prediction (Supplementary information, Figure S3). The L2 region is predicted to form an α-helix, which is connected on its C terminal end to a helix region of the HK domain. Figure 4b shows the position of the N-terminal end of each truncated L2 region in the α-helical wheel model, and their assigned gene regulation pattern category (Types 1 to 4). The members of each category are all grouped together on the α-helical wheel model. Most remarkably, Type 4 miniaturized CcaSs, which did not show any gene induction, are all located on the opposite side of α-helix from Types 1 to 3, which all show gene induction. Interestingly, Type 1, showing gene induction under green light, and Type 3, showing gene induction under red light, were divided by the Type 2, which showed gene induction under both conditions. Considering that YF1 and miniaturized CcaSs constructed in this study have similar domain structures (the light sensor and HK domains), our truncation study suggests a similar mechanism of CcaS photoregulation with that of YF1, which follows rotary-switch mechanism.

Another feature of the miniaturized CcaSs is their ability to tightly regulate gene expression, achieving high expression of the target gene under inductive conditions and strict gene repression under non-inductive condition. The most prominent structural difference between wild-type CcaS and miniaturized CcaSs is the deletion of the PAS domains. Therefore, the removal of the PAS domains is likely the main factor behind the achieved tight gene regulation. The current experimental results were limited to demonstrating the function of the engineered CcaSs in *E. coli* as a host microorganism. However, this study has demonstrated the potential for future engineering of this light regulated gene expression system, as long as the engineered CcaS is expressed as a soluble protein with a chromophore to show light dependent activation/inactivation of CcaR. In order to evaluate the function of the engineered CcaSs in cyanobacteria, the preparation of a *ccaS* deletion mutant of PCC 6803, or replacement of the endogenous *ccaS* gene with the engineered *ccaS* gene in the PCC 6803 genome, will be inevitable. The functional expression of the engineered CcaSs in cyanobacteria is highly expected, since the CcaS N-terminal membrane binding region, which is essential to localize this photoreceptor in cyanobacteria, was not modified.

In conclusion, we developed miniaturized CcaSs that showed high gene expression regulation ability by truncation of two PAS domains. These miniaturized CcaSs have a high potential for future application in the light regulated gene expression-based bioprocesses, which have significant advantages compared with chemical induction. Notably, the regulation of cellular processes by green and red light enable the modulation of photosynthesis-dependent biotechnological processes, which require reducing equivalents. In regards to growth in closed photobioreactors under artificial light, electrons originating from photosynthetic water-splitting may partly be re-routed for the reduction of valuable carbon-compounds^26^ or for the direct production of biohydrogen, especially in engineered cyanobacterial cells^27^ upon red or green light induction. As this can happen in a time-dependent manner, such light-triggered processes add a new dimension not only to the engineering of cyanobacterial cells for technical applications but also for non-photosynthetic microorganisms based processes.

## Material and Methods

### Sequence Analysis and Modeling the Three-Dimensional Structure of Histidine Kinase Domains of CcaS

Sequences of HK domains of CcaS (residue 504–753) and YF1 (residue 129–245) were aligned using ClustalW^28^. Secondary structure predictions of CcaS were performed using the Jpred program^29^. We then performed homology modeling with MODELLER version 9.16 30 using the crystal structure of YF1 (PDB code 4gcz^24^) as a template.

#### Construction of Vectors

**Figure 5:**
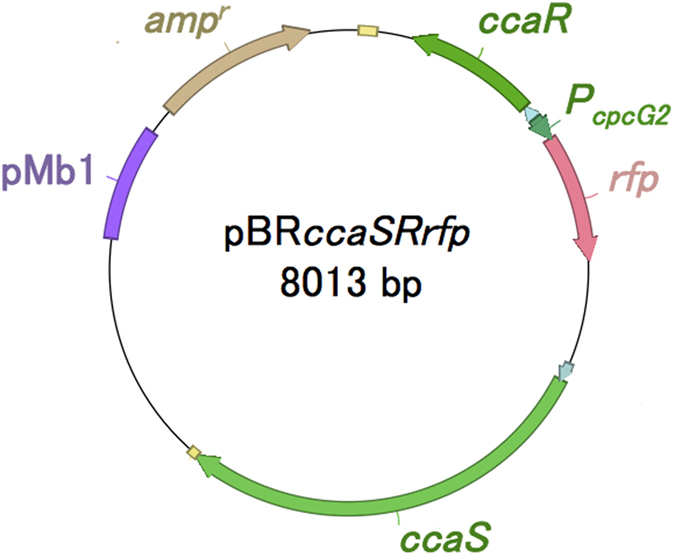
Constructed plasmid. pBR*ccaSRrfp* encoding the light sensor HK CcaS, the cognate response regulator CcaR, and RFP as a reporter protein under the control of the P*cpcG2* promoter.

The *rfp* gene from the high-copy-vector pSB1A3 (from the BioBrick Registry of Standard Biological Parts (http://partsregistry.org)) was inserted into the *Xba*I and *Nde*I sites of pKTGSS^22^. The green-light-sensing system coding region containing *rfp* gene under the P_*cpcG2*_ promoter was amplified by polymerase chain reaction (PCR) with the following primers: 5′-AGCGGCCGCGAATTCTTGAAGACGAAAGGGCCTC-3′ and 5′-TTTTTTCGCCTGCAGATGGAAGCCGGCGGCAC-3′. The section of pBR322 31 without the *tetC* coding region was amplified with the primers 5′-GAATTCGCGGCCGCTTCTAG-3′ and 5′-CTGCAGGCGAAAAAACCCCGCCGAAG-3′. These two PCR products were ligated by In-Fusion Cloning Kit (Takara Bio Inc., Otsu, Shiga, Japan) and the resulting plasmid was named pBR*ccaSRrfp* (Fig. 5).

The section of pBR*ccaSRrfp* without the region encoding the two PAS domains of CcaS gene was amplified by PCR using distinct primer sets for the construction of each CcaS mutant. The primers used are shown in Supporting Data, TableS1. The end of each linearized plasmid was phosphorylated using polynucleotide T4 kinase (Takara Bio Inc., Otsu, Shiga, Japan) and ligated using Takara ligation kit according to the manual (Takara Bio Inc., Otsu, Shiga, Japan). They were used for transformation of *E. coli* DH5α. The plasmids are constructed by inserting *CcaS* gene mutants in pBR*ccaSRrfp*, instead of gene encoding the wild-type CcaS (Supporting Data, Table S2).

The region of pBR*ccaSRrfp* without *rfp* gene was amplified by PCR. The PCR products were self-ligated as described above. The resulting plasmid named pBR*ccaSR*no*rfp* was used as a negative control.

#### Characterization of CcaS mutants

Transformants of *E. coli* DH5α harboring the plasmid encoding the light sensing system and the plasmid pSTVPCB, which encodes PCB synthesis gene cassette previously constructed^5^, were cultured in a 700 μL LB broth including appropriate antibiotics using a 96-deep well plate (Thermo Fisher Scientific, Waltham, MA, USA) sealed by Breathe-Easy^®^ sealing membrane (Sigma Aldrich, St. Louis, MO, USA) at 37 °C using a bioshaker (Biomedical Science, Shinjuku, Tokyo, Japan) at 1000 rpm for 16 hours under dark conditions. The cultures were inoculated to a fresh 700 μL LB broth including appropriate antibiotics. Two cultures in triplicate for each transformant were cultured in a 96-deep wells plate as described above. *Escherichia coli* transformants were grown under red light (660 nm, 40 μmol m^−2^ s^−1^) or green light (520 nm, 40 μmol m^−2^ s^−1^) illumination separately for 17 hours. Next, 50-μL cultures of each transformant were transferred to a clear 96-well plate (Thermo Fisher Scientific, Waltham, MA, USA) and 150 μL fresh media was added to each culture. The cell density of the cultures was monitored by measuring the optical density at 595 nm (OD_595_) in a plate reader (Thermo Fisher Scientific, Waltham, MA, USA).

The cultures were centrifuged and supernatant was discarded. The culture pellets were then resuspended in 300 μL of phosphate-buffered saline (PBS). Then, 250-μL suspension was transferred to a black 96-well plate (Thermo Fisher Scientific, Waltham, MA, USA). The fluorescence intensity of the suspension was measured using a plate reader (Thermo Fisher Scientific, Waltham, MA, USA) with excitation and emission wavelengths of 584 nm and 607 nm, respectively. To evaluate the amount of protein expression induced by the light sensing system, fluorescence per cell was calculated. These procedures were triplicated using newly prepared transformants for each experiment.

#### Monitoring of Green or Red Light-Regulated Gene Expression Using Miniaturized CcaSs

The investigation for each miniaturized CcaS was carried out by a single fluorescence measurement performed on triplicate cultures of a single transformant. In detail, transformants of *E. coli* DH5α harboring miniaturized CcaS were cultured in 2 mL of LB broth, including appropriate antibiotics, for 12 hours at 37 °C under dark. The cultures were inoculated to dilute to OD_595_ = 0.02 in 20 mL of fresh LB broth, including appropriate antibiotics, in a 50-mL Erlenmeyer flask in triplicates. Two cultures set in triplicates of each transformant were cultured at 37 °C under non-inductive condition while being shaken at 100 rpm for 4 hours. Then, one of these culture sets in triplicates were cultured at 37 °C under inductive conditions while being shaken at 100 rpm. The other set continued to be cultured in the same manner under non-induced conditions.

During culturing, 50 μL of the cultures were sampled periodically to a clear 96-well plate, 200-μL fresh media was added to each culture, and then cell densities were measured by plate reader. At the same time, 700 μl of the cultures were sampled to a 96-well deep well plate. The cultures were centrifuged at 12,000 g for 2 min at 25 °C and the supernatant was discarded.

Each cell pellet was resuspended in PBS, then centrifuged. Again each resulting cell pellet was resuspended in 200 μL PBS and transferred to a black 96-well plate. The RFP fluorescence of each cell was measured by a plate reader with excitation and emission wavelengths of 584 nm and 607 nm, respectively. Then 20 μL of each cell suspension was transferred to a clear 96-well plate and diluted by adding 180 μL PBS. Next, cell density was analyzed by measuring OD_595_ using a plate reader.

## Additional Information

**How to cite this article**: Nakajima, M. *et al.* Construction of a Miniaturized Chromatic Acclimation Sensor from Cyanobacteria with Reversed Response to a Light Signal. *Sci. Rep.* **6**, 37595; doi: 10.1038/srep37595 (2016).

**Publisher’s note:** Springer Nature remains neutral with regard to jurisdictional claims in published maps and institutional affiliations.

## Supplementary Material

Supplementary Information
