# Supplementary material for: Construction of a Miniaturized Chromatic Acclimation Sensor from Cyanobacteria with Reversed Response to a Light Signal
Source: Sci Rep. 2016 Nov 24;6:37595. doi: 10.1038/srep37595 (PMC5121610; doi:10.1038/srep37595)
Supplement: Supplementary Information [file srep37595-s1.pdf]

# Construction of a Miniaturized a Chromatic Acclimation Sensor from Cyanobacteria with Reversed Response to a Light Signal

Mitsuharu Nakajima[1, 2], Stefano Ferri[2, 3], Matthias Rögner[4], *Koji Sode[1, 2, 5]

Key words; green light induction, green light sensor, optogenetics, *Escherichia coli*

[1]Department of Biotechnology and Life Science, Graduate School of Engineering, Tokyo University of Agriculture & Technology, 2-24-16 Naka-cho, Koganei, Tokyo 184-8588, Japan

[2]JST, CREST, 2-24-16 Naka-cho, Koganei, Tokyo 184-8588, Japan

[3] Department of Applied Chemistry and Biochemical Engineering, Shizuoka University, 3-5-1 Johoku, Naka-ku, Hamamatsu, Shizuoka, 432-8561, Japan

[4] Plant Biochemistry, Faculty of Biology & Biotechnology, Ruhr-Universität Bochum, Universitätsstr. 150, 44780 Bochum, Germany

[5]Institute of Global Research Innovation, Tokyo University of Agriculture & Technology, 2-24-16 Naka-cho, Koganei, Tokyo 184-8588, Japan

*Corresponding author

Correspondence: sode@cc.tuat.ac.jp

**Supplementary Table S1** Vectors used in this study.

| lasmid name | Origin | Resistance | Features | Source |
|---|---|---|---|---|
| pBR*ccaSR*no*rfp* | pMb1 ori | Ampicillin | Construct for expressing CcaS, CcaR, and *cpcG2* promoter | This study |
| pBR*ccaSRrfp* | pMb1 ori | Ampicillin | Construct for expressing CcaS, CcaR, and RFP transcriptionally driven by *cpcG2* promoter | This study |
| pBR*ccaS*#1 | pMb1 ori | Ampicillin | Construct for expressing CcaS#1, CcaR, and RFP transcriptionally driven by *cpcG2* promoter | This study |
| pBR*ccaS*#2 | pMb1 ori | Ampicillin | Construct for expressing CcaS#2, CcaR, and RFP transcriptionally driven by *cpcG2* promoter | This study |
| pBR*ccaS*#3 | pMb1 ori | Ampicillin | Construct for expressing CcaS#3, CcaR, and RFP transcriptionally driven by *cpcG2* promoter | This study |
| pBR*ccaS*#4 | pMb1 ori | Ampicillin | Construct for expressing CcaS#4, CcaR, and RFP transcriptionally driven by *cpcG2* promoter | This study |
| pBR*ccaS*#5 | pMb1 ori | Ampicillin | Construct for expressing CcaS#5, CcaR, and RFP transcriptionally driven by *cpcG2* promoter | This study |
| pBR*ccaS*#6 | pMb1 ori | Ampicillin | Construct for expressing CcaS#6, CcaR, and RFP transcriptionally driven by *cpcG2* promoter | This study |
| pBR*ccaS*#7 | pMb1 ori | Ampicillin | Construct for expressing CcaS#7, CcaR, and RFP transcriptionally driven by *cpcG2* promoter | This study |
| pBR*ccaS*#8 | pMb1 ori | Ampicillin | Construct for expressing CcaS#8, CcaR, and RFP transcriptionally driven by *cpcG2* promoter | This study |
| pBR*ccaS*#9 | pMb1 ori | Ampicillin | Construct for expressing CcaS#9, CcaR, and RFP transcriptionally driven by *cpcG2* promoter | This study |
| pBR*ccaS*#10 | pMb1 ori | Ampicillin | Construct for expressing CcaS#10, CcaR, and RFP transcriptionally driven by *cpcG2* promoter | This study |
| pBR*ccaS*#11 | pMb1 ori | Ampicillin | Construct for expressing CcaS#11, CcaR, and RFP transcriptionally driven by *cpcG2* promoter | This study |
| pKTGSS | V ori, p15A ori | Streptomycin | Construct for expressing CcaS, CcaR, and GFPuv transcriptionally driven by *cpcG2* promoter | [22] |
| pBR322 | pMb1 ori | Ampicillin/tetracycline | Original vector used for construction of pBRGLAg | [31] |
| pSTVPCB | p15A ori | Chloramphenicol | *ho1* and *pcyA* genes for PCB synthesis | [5] |

**Supplementary Table S2** Primers used for the construction of CcaS mutants.

| Primer name | Sequence |
|---|---|
| #Nrev | 5'-CTGTAATTGCTCATATAATTCCGATTG-3' |
| #1fw | 5'-GAGCGCCGACGCACG-3' |
| #2fw | 5'-CGCCGACGCACGGAG-3' |
| #3fw | 5'-CGACGCACGGAGGAAGTC-3' |
| #4fw | 5'-CGCACGGAGGAAGTCCG-3' |
| #5fw | 5'-ACGGAGGAAGTCCGCC-3' |
| #6fw | 5'-GAGGAAGTCCGCCTAGCTTTAG-3' |
| #7fw | 5'-GAAGTCCGCCTAGCTTTAGAAC-3' |
| #8fw | 5'-GTCCGCCTAGCTTTAGAACG-3' |
| #9fw | 5'-CGCCTAGCTTTAGAACGGG-3' |
| #10fw | 5'-CTAGCTTTAGAACGGGAAAAAGAATTAAG-3' |
| #11fw | 5'-GCTTTAGAACGGGAAAAAGAATTAAG-3' |

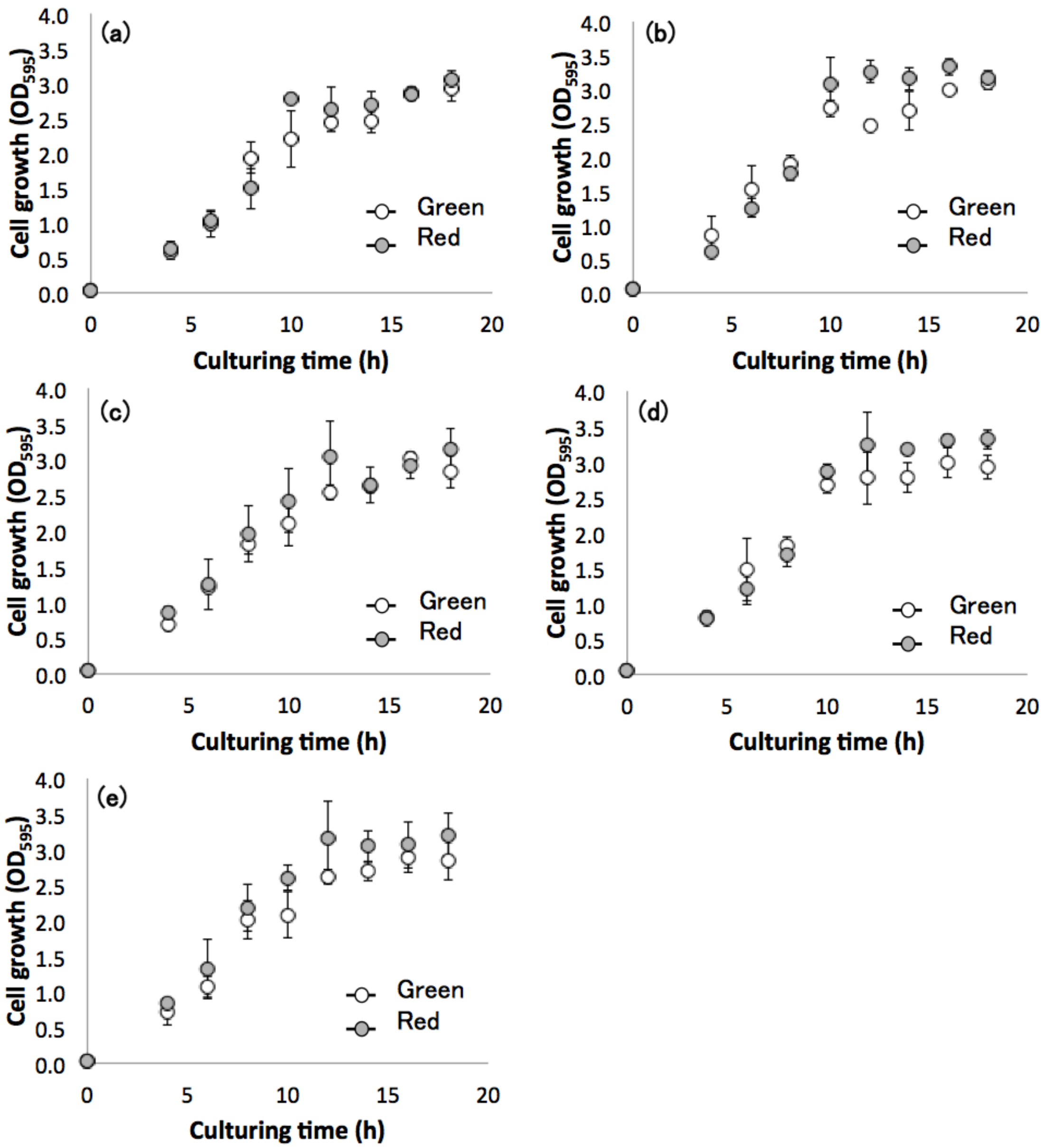


**Supplementary Figure S1** Growth of cells harboring light sensing systems with CcaS variants. Cell growth was monitored of cells cultured under either green (white circles) or red (gray circles) light exposure. Growth of cells harboring a light sensing system with CcaS wild type and without *rfp* gene (a). Growth of cells harboring the *rfp* gene and a light sensing system with either the wild-type CcaS (b), CcaS #3 (c), CcaS #10 (d), or CcaS #11(e). Data represent means ± SD from independent triplicate experiment from one clone (three experiments).

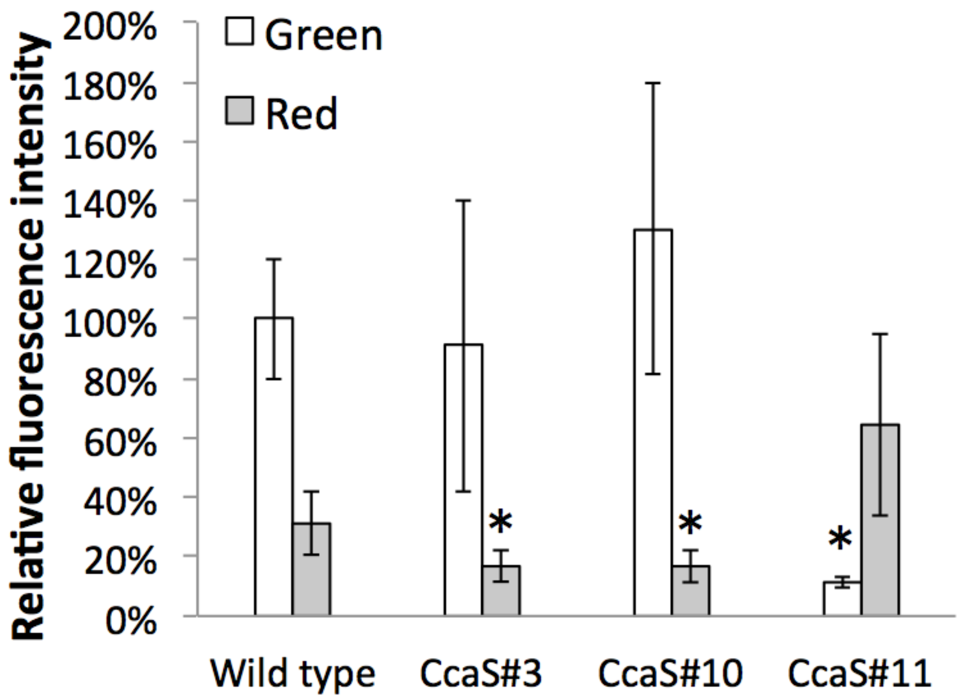


**Supplementary Figure S2** The comparisons of RFP expression level under non-induced conditions (red light; wild type, CcaS#3, CcaS#10, green light; CcaS#11), and induced conditions (green light; wild type, CcaS#3, CcaS#10, red light; CcaS#11) for each miniaturized CcaS. Bars show the RFP fluorescence of cells grown under green light (white bars) or red light (gray bars). Bars indicate relative fluorescence intensity to normalized fluorescence level of wild type in each experiment using three different transformants. GFP fluorescence under induced conditions were sampled at the time when the highest fluorescence observed (12 hours; wild type, CcaS#3, CcaS#10, 14 hours; CcaS#11). Each three experiment were performed in triplicate. Data represent means ± SD from independent triplicate experiment from each of three clones (nine experiments). Asterisks indicate statistically significant differences between fluorescence of cells harboring wild type under non-inductive condition and of cells harboring CcaS mutants under non-inductive condition (Dunnet's test; *$P < 0.001$).

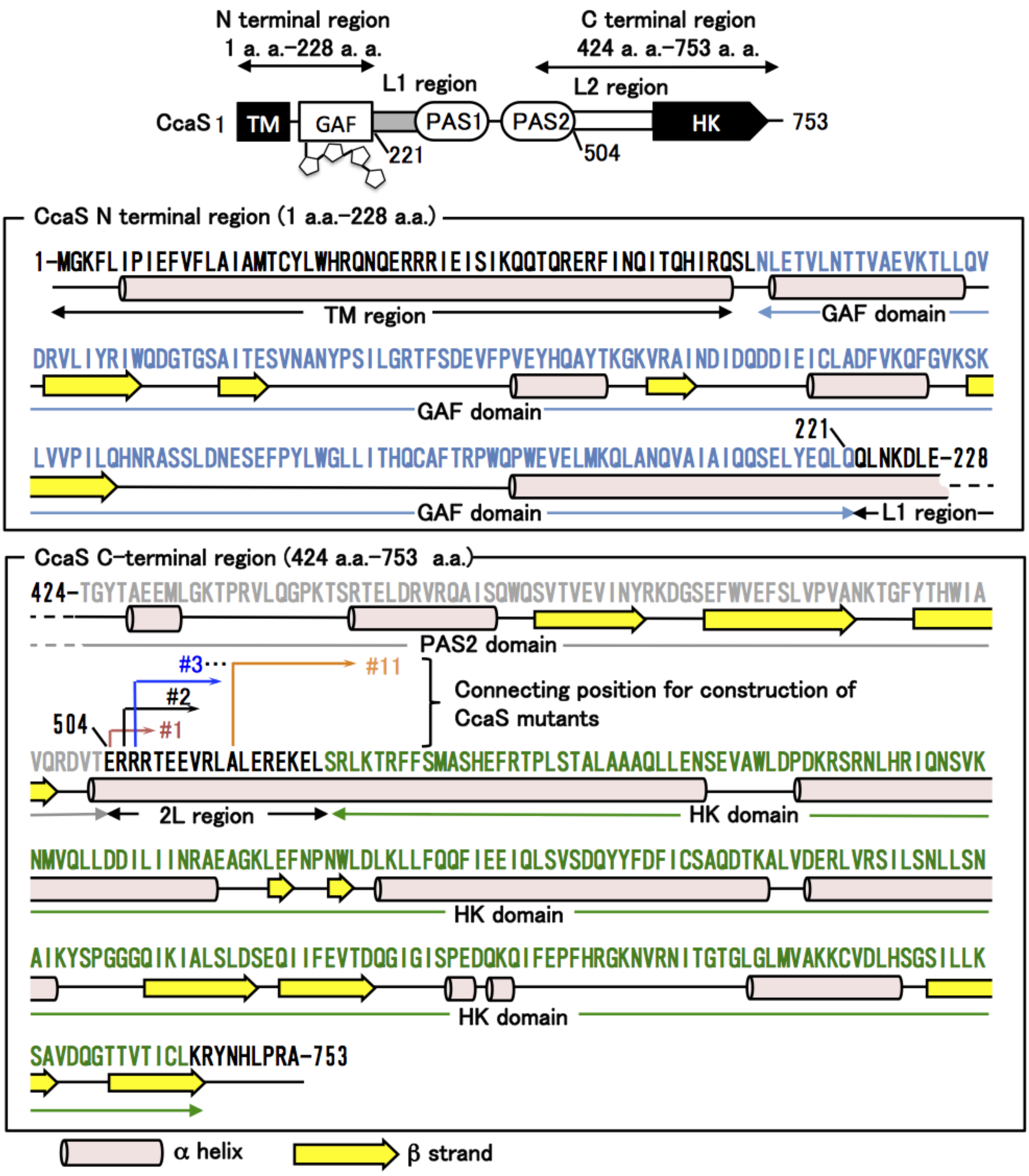


**Supplementary Figure S3** Secondary structure prediction of the N-terminal (Met1 to Glu228) and C-terminal (Thr424 to Ala753) regions of wild-type CcaS.
